# Cultivation of *Rickettsia amblyommii* in tick cells, prevalence in Florida lone star ticks (*Amblyomma americanum*)

**DOI:** 10.1186/1756-3305-7-270

**Published:** 2014-06-13

**Authors:** Katherine A Sayler, Heather L Wamsley, Melanie Pate, Anthony F Barbet, A Rick Alleman

**Affiliations:** 1 Department of Physiological Sciences, University of Florida College of Veterinary Medicine, Gainesville, Florida, USA; 2 Department of Infectious Diseases and Pathology, University of Florida College of Veterinary Medicine, Gainesville, Florida, USA

**Keywords:** *Amblyomma americanum*, Lone star tick, *Rickettsia amblyommii*, Tick-borne, Tick cell culture

## Abstract

**Background:**

*Rickettsia amblyommii* is a bacterium in the spotted fever group of organisms associated with the lone star tick (LST), *Amblyomma americanum*. The LST is the most commonly reported tick to parasitize humans in the southeastern US. Within this geographic region, there have been suspected cases of Rocky Mountain spotted fever (RMSF) where the causative agent, *R. rickettsii,* was not identified in the local tick population. In these areas, patients with clinical signs of RMSF had low or no detectable antibodies to *R. rickettsii*, resulting in an inability to confirm a diagnosis*.*

**Methods:**

*R. amblyommii* was cultivated from host-seeking LSTs trapped in Central Florida and propagated in ISE6 (*Ixodes scapularis*) and AAE2 (*A. americanum*) cells. Quantitative PCR targeting the 17-kD gene of *Rickettsia* spp. identified the genus of the organism in culture. Variable regions of *gro*EL, *gtl*A and *romp*A genes were amplified and sequenced to confirm the species. The prevalence of *R. amblyommii* in LSTs within the geographic region was determined by qPCR followed by conventional PCR and direct sequencing.

**Results:**

Analyses of amplified sequences from the cultured organism were 100% homologous to *R. amblyommii*. The overall prevalence of *Rickettsia* spp. in the local population of LSTs was 57.1% and *romp*A sequence analysis identified only *R. amblyommii* in LSTs.

**Conclusions:**

A Florida strain of *R. amblyommii* was successfully cultivated in two tick cell lines. Further evaluation of the new strain and comparisons to the other geographic strains is needed. The prevalence of this SFG organism in the tick population warrants further investigation into the organism’s ability to cause clinical disease in mammalian species.

## Background

*Rickettsia amblyommii* is an incompletely characterized alphaproteobacterium in the spotted fever group (SFG) of organisms. This organism has been identified in many species of *Amblyomma* ticks including *Amblyomma americanum, Amblyomma maculatum* and *Amnlyomma cajennense.* The preferred tick host in the United States appears to be the lone star tick (LST), *A. americanum,* and has been identified in 41.2% [1] to 64.5% [2] of host-seeking *A. americanum* collected from diverse geographic regions of the United States. The organism was reportedly highly prevalent (89%) in pools of the immature life stages of *A. americanum* by polymerase chain reaction (PCR) [3] and was detected in 66.5% to 80.5% of *A. americanum* pools that were directly parasitizing humans [4]. The pervasiveness of this bacterium in all life stages of the vector and in both host-seeking and partially fed ticks, underscores the possibility that it could be transmitted to a mammalian host, as all life stages of the LST will parasitize large mammals [5].

*A. americanum* is a promiscuous, aggressive feeder long known to be a pest and, more recently, a vector of multiple zoonotic pathogens [6-9]. Rocky Mountain spotted fever (RMSF) is most notably caused by *Rickettsia rickettsii,* which is primarily transmitted by *Dermacentor variabilis* and *Dermacentor andersoni* ticks in the United States*.* However, *R. amblyommii* has been implicated as a cause of RMSF-like disease in humans [10] and has been associated with a rash following an *A. americanum* bite [11]. In addition, cases of RMSF are commonly reported in bands across the southeastern United States [12] that correspond to the geographic range of *A. americanum*, even in areas in which *Dermacentor variabilis* infected with *R. rickettsii* are low in number or absent. This complicates the diagnosis of RMSF in areas where *A. americanum* is the predominant tick species parasitizing humans [10,13,14]. In these areas, it has been suggested that reports of RMSF are more likely due to other *Rickettsia* spp., such as *R. amblyommii*[15,16]. However, due to *R. amblyommii’s* symbiont-like pervasiveness and lack of culture isolation from a mammalian species, the importance of this bacterium as a human or animal pathogen is still unclear.

The development of culture systems for the growth of *Rickettsia* is critical to the genetic and antigenic evaluation of pathogenic and nonpathogenic species, and for comparison of isolates from different geographic regions. It has been shown that growth of tick-borne organisms in different cell lines results in variable expression of important antigens [17]. Because cultured organisms may vary antigenically depending on the culture system used, it would be most beneficial to propagate organisms in cell lines derived from the respective vectors. In the United States, *R. amblyommii* has been propagated in the mosquito *Anopheles gambiae* cell line [2], and in South America and Central America, African green monkey kidney (Vero) cells have been used to propagate *R. amblyommii* from *A. cajennense* ticks [18]. The growth of *R. amblyommii* in a cell line derived from the natural vector, *A. americanum*, could facilitate the generation of large numbers of bacteria for genomic, proteomic, diagnostic, and experimental studies [19]. The development of diagnostic assays specific for *R. amblyommii* infection is needed, as current serology-based methods lack specificity due to cross-reactive antigens within the RMSF group [14].

In this study, *A. americanum* (AAE2) and *Ixodes scapularis* (ISE6) cell lines were used to culture *R. amblyommii* from wild *A. americanum* specimens that were trapped in North Central Florida. The prevalence rate of *R. amblyommii* in over 1,400 adult, host-seeking *A. americanum* specimens was also determined using PCR analysis of individually dissected ticks. To our knowledge, this is the first report of successful propagation of *R. amblyommii* in an *A. americanum* tick cell line.

## Methods

### Tick collection

**Figure 1:**
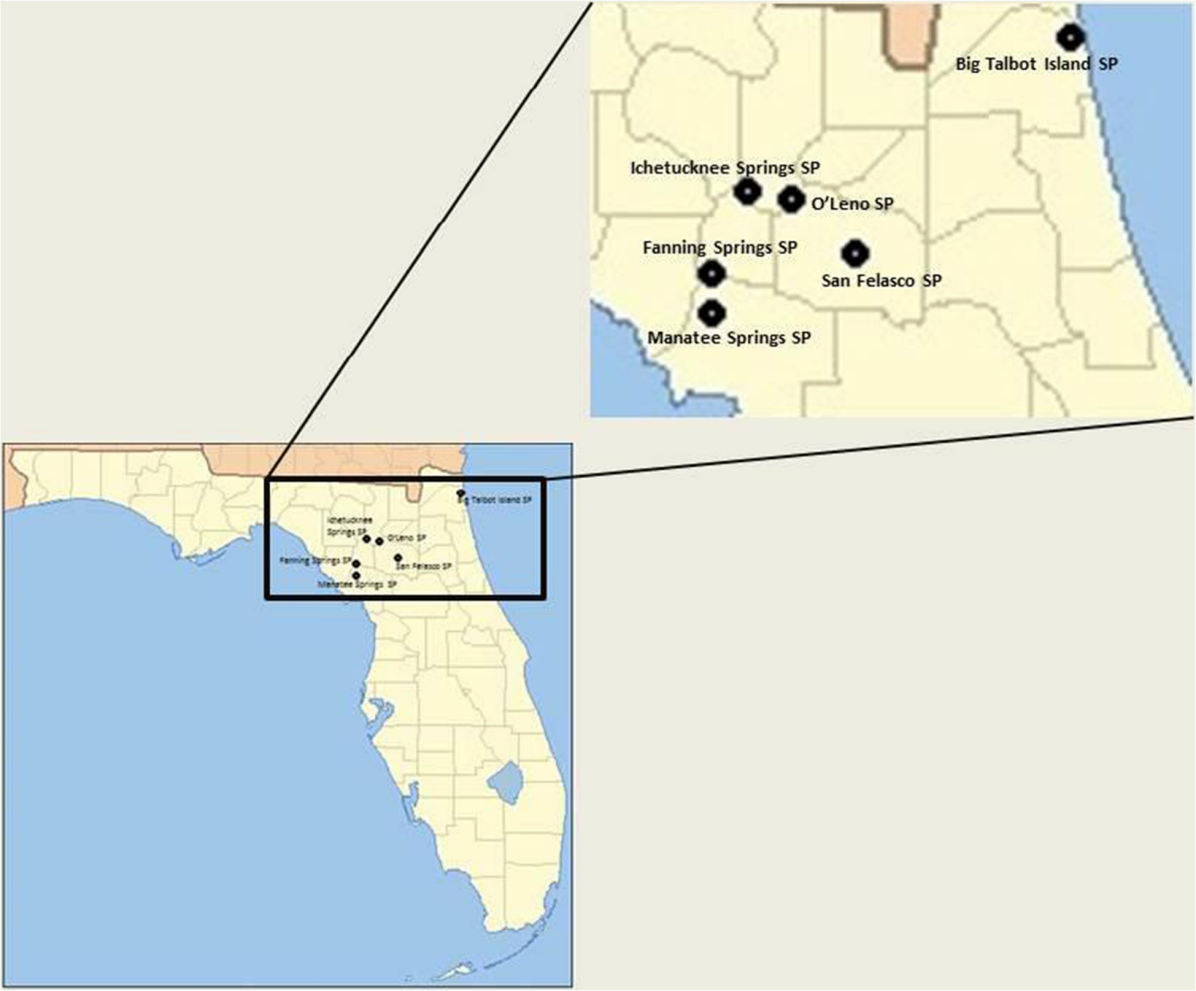
Map of collection sites at 5 state parks in North Central Florida.

A total of 1,479 host-seeking, unengorged, adult *A. americanum* ticks were collected from six state parks in North Central Florida (Figure 1), during the months of May through September of 2010 and 2011 and March through August of 2012. At sites where ticks were collected on multiple occasions (O’Leno, San Felasco and Manatee Springs), multiple sites were sampled, typically around wooded areas adjacent to the parking lots, playground equipment, and the main campgrounds of each facility. Ticks were collected according to guidelines of the Florida Department of Environmental Protection research and collection permits #05201020-A, 06201120 and 06201120, and in agreement with the Florida Division of Recreation and Parks. Briefly, ticks were collected using CO_2_-emitting dry-ice traps as previously described [20] and by flagging. Flagging was accomplished by dragging a 1 meter × 1.5 meter white linen cloth over areas of high grass and brush. Live ticks were transported in double-sealed containers in designated vehicles to the University of Florida, College of Veterinary Medicine (CVM) for processing. Viable *A. americanum* were identified and sexed according to published guidelines and stored at 4°C in the transport containers until prepared for dissection (no more than 4-week storage time). Individual ticks were dissected under aseptic conditions in sterile petri dishes using flame-sterilized scalpels and dissection forceps. Prior to dissection, each tick was washed in 200 μL of a 10% bleach solution for five minutes, followed by three washes in 200 μl of 70% ethanol, vortexed for 15 seconds per wash in a DNA-free SealRite PCR tube (USA Scientific, Ocala, Florida), and then rinsed three times in deionized water to remove residual alcohol. Midguts and salivary glands were removed from each tick and placed singly in 200 μl of sterile 1X phosphate buffered saline (Cellgro, Mediatech Inc., Manassas,Virginia). Genomic DNA was extracted from the dissected material using DNeasy Blood and Tissue Kit (Qiagen,Valencia, California) according to the manufacturer’s instructions. Tick dissections and gDNA extractions were performed in separate, designated areas of the laboratory to maintain a clean environment and sterile petri dishes, instruments, and filtered tips were used in every step of tick preparation for molecular work. In addition, four pools of 15 *A. americanum* specimens were similarly dissected for immediate inoculation into cell cultures.

### PCR

Real-time PCR targeting a conserved region of the 17-kD antigen gene of *Rickettsia* spp. was performed using primers R17D135F and R17D249R and a dual-labeled Taqman probe as previously described [21]. Individual DNA samples were tested in MicroAmp Fast Optical 96-well reaction plates (Applied Biosystems, Foster City, California), with 2 μL of template DNA in a 20 μl final reaction volume. PCR amplification and data analysis were performed using the Prism 7500 thermocycler and associated software (Applied Biosystems, Foster City, California). Brilliant II QPCR Master Mix (Strategene, La Jolla, California) was used for all reactions under the following thermocycler conditions: 95°C for 10 minutes, followed by 40 cycles of 95°C for 15 seconds and 57°C for 60 seconds. To further characterize organisms in *Rickettsia*-positive ticks, the *romp*A gene was targeted in a 50 μl conventional PCR reaction, as previously described [22]. Amplicons were identified on a 2% tris-acetate-EDTA (TAE) gel by electrophoresis and visualized under UV light using Gelstar nucleic acid gel stain (Lonza, Rockland, Maine). PCR products were purified using QIAquick PCR purification kit (Qiagen, Valencia, California) according to the manufacturer’s instructions. Purified amplicons were sequenced at the University of Florida Interdisciplinary Center for Biotechnology Research (ICBR) core laboratory. In both protocols described above, *Rickettsia conorii*, which was graciously provided by Dr. Jere McBride and Dr. Xuejie Yu (Center for Biodefense and Emerging Infectious Diseases; Sealy Center for Vaccine Development, University of Texas Medical Branch, Galveston, Texas) was used to generate standards and controls for *Rickettsia* spp. Nucleic acid-free molecular grade water was used as a negative control in all PCR reactions and PCR was performed in a designated area in a laboratory, separate from the area in which culture of organisms took place.

### Source and preparation of *R. amblyommii* inocula

An additional 60 adult, female, host-seeking *A. americanum* were collected from O’Leno State Park in High Springs, Florida, in June 2012. Live LSTs were transported to the University of Florida CVM in double-sealed containers and stored overnight at 4°C. The following morning, ticks were washed and dissected under sterile conditions as described above, and pooled into 4 groups of 15 ticks each. Pools were transferred to tubes containing 1 mL of cold tick cell culture medium L-15B300 [23] supplemented with 20% fetal bovine serum (FBS) (Sigma, St. Louis, Missouri), and penicillin, streptomycin, and amphotericin B (50 IU/mL, 50 μg/mL, and 0.125 μg/mL, respectively) (Antimicrobial Solution 17–745, Lonza BioWhittaker, Basel, Switzerland). Preparations were maintained at 4°C to maintain organism viability.

### Cultivation of *R. amblyommii* in tick cell culture

Two tick cells lines (*Amblyomma americanum*, AAE2 and *Ixodes scapularis*, ISE6)^a^ were cultivated in T25 flasks as described elsewhere [24]. When cells were confluent, 4 flasks (1 flask containing AAE2, and 3 flasks containing ISE6) were each inoculated with a pooled dissected-tick preparation described above. Immediately after inoculation, the flasks were incubated (25˚C) in a biological safety cabinet for 30 minutes. Subsequently, the flasks were brought to full volume (7 mL) in L-15B300 medium supplemented with FBS, penicillin, streptomycin, and amphotericin B as described above. Flasks were maintained unvented at 34˚C with weekly medium changes and passages as needed. The antimicrobial solution was no longer added to the medium after week 4 of cultivation. Flasks were monitored for infection using phase contrast microscopy and Wright-Giemsa stained cytocentrifuged preparations of detached cells were examined using light microscopy.

### Identification of cultivated organisms

Infected tick cells were detached from culture flasks using a cell scraper or pipette fluid stream, transferred in medium to a 1.5 mL microcentrifuge tube and centrifuged at 13,000 rpm for 10 minutes. The cell pellet was resuspended in 100 μL of sterile 1X phosphate buffered saline (PBS). Genomic DNA was isolated from cell cultures using DNeasy Blood and Tissue Kit cell culture protocol (Qiagen, Valencia, California) and gDNA was quantified using fluorometry (Qubit, Invitrogen, Carlsbad, California). A minimum of 150 ng of DNA was screened for the presence of *Rickettsia* sp., targeting the 17-kD conserved region as outlined above, and species identification was performed using primers that target the *romp*A gene, as outlined above. Purified amplicons were sequenced and analyzed as described above.

Additional primer sets were also used to confirm the identity of the cultured *Rickettsia* sp. The *gro*EL gene of SFG *Rickettsia* sp. was targeted in conventional PCR as previously described [25] slightly modified by employing Taq PCR Master Mix (Qiagen, Valencia, California) under the following conditions: 95°C for 3 minutes, followed by 30 cycles of 94°C for 30 seconds, 56°C for 30 seconds, 72°C for 30 seconds, with a 72°C final extension for 5 minutes. Additionally, the citrate synthase gene (*glt*-A) was also targeted as previously described [26] using primers CS-78 and CS-323, for specific genetic identification of the infecting rickettsiae. All PCR products of the expected size were visualized, purified, sequenced and analyzed as outlined above. To confirm that other species of bacteria were not present in culture, the 16s rRNA gene was targeted using bacteria-wide primers, followed by the sequencing of individual clones (n = 36) using the TOPO-4 vector system as previously described [27].

Rickettsial organisms were visualized using light microscopy and photomicrographs were captured using digital photography (Olympus BH2, Olympus, Tokyo, Japan).

## Results and discussion

### Infection survey

In 2010, a total of 207 unfed adult *A. americanum* specimens from four counties in central Florida were surveyed (Table 1). The frequency of *R. amblyommii* ranged from 39.8% to 75% of individual ticks per state park collection site. Overall, 44.0% of surveyed ticks carried detectable levels of *R. amblyommii.* In 2011, a total of 908 unfed, host-seeking *A. americanum* specimens were trapped and screened for the presence of *R. amblyommii* (Table 2). Frequency of the organism ranged from 49.2% to 66.7% of individual ticks collected per collection site. Overall, 50.8% of ticks surveyed in 2011 carried detectable levels of *R. amblyommii.* In 2012, the investigators collected 364 ticks from two parks in which *A. americanum* populations were highest in 2010, particularly in heavily wooded areas (Table 3). The frequency of *R. amblyommii* ranged from 47.1% to 93.5% of the individual ticks by collection site. Overall, 80.5% of the ticks surveyed in 2012 carried detectable levels of *R. amblyommii*. The average prevalence of *Rickettsia* in all parks over the three-year collection period was 57.1% of individual host-seeking LSTs (845/1479). This is similar to previously reported prevalence of the organism in LSTs in other geographic areas in the southeastern United States [1]. *R. amblyommii* was the only *Rickettsia* spp. detected in all LSTs collected over the three-year period in North Central Florida using the methodology of PCR targeting the *romp*A gene followed by Sanger sequencing. Our findings are similar to those of other studies that tested for rickettsial infections in the United States in that no other rickettsial species were found in adult host-seeking LSTs [10,14]. These data suggest that potential exposure to this organism from the bite of a LST is high. This is of particular concern because all stages of the LST feed on humans, especially adults [3]. Furthermore the tick is expanding its endemic range and increasing its population in established areas, putting people at risk for contacting a potentially infected tick [4].

**Table 1:** 2010 LST collection data by Florida state park location

**State park name and location**	**Primary month of collection**	**n**	**Detectable *R. amblyommii *** **confirmed by sequencing**
O’Leno, Columbia Co.	June	133	53 (39.8%)
San Felasco, Alachua Co.	July, August	53	24 (45.3%)
Manatee Springs, Levy Co.	September	16	12 (75.0%)
Ichetucknee, Columbia Co.	July	5	2 (40.0%)
	**TOTAL:**	207	91 (44.0%)

The total number of *R. amblyommii*-positive *A. americanum* specimens are given in the final column, with percent infected in parentheses.

**Table 2:** 2011 LST collection data by Florida state park location

**State park name and location**	**Primary month of collection**	**n**	**Detectable *R. amblyommii *** **confirmed by sequencing**
O’Leno, Columbia Co.	May, June	391	200 (51.2%)
San Felasco, Alachua Co.	June	472	232 (49.2%)
Manatee Springs, Levy Co.	June	12	8 (66.7%)
Fanning Springs, Gilchrist Co.	June	33	21 (63.6)
Big Talbot Island, Duval Co.	July	0	0
	TOTAL:	908	461 (50.8%)

The total number of *R. amblyommii*-positive *A. americanum* specimens are given in the final column, with percent infected in parentheses.

**Table 3:** 2012 LST collection data by Florida state park location

**State park name and location**	**Primary month of collection**	**n**	**Detectable *R. amblyommii *** **confirmed by sequencing**
O’Leno, Columbia Co.	April, May	262	245 (93.5%)
San Felasco, Alachua Co.	June	102	48 (47.1%)
	TOTAL:	364	293 (80.5%)

The total number of R. *amblyommii*-positive *A. americanum* specimens are given in the final column, with percent infected in parentheses.

### Isolation and culture of *R. amblyommii* in tick cells

**Figure 2:**
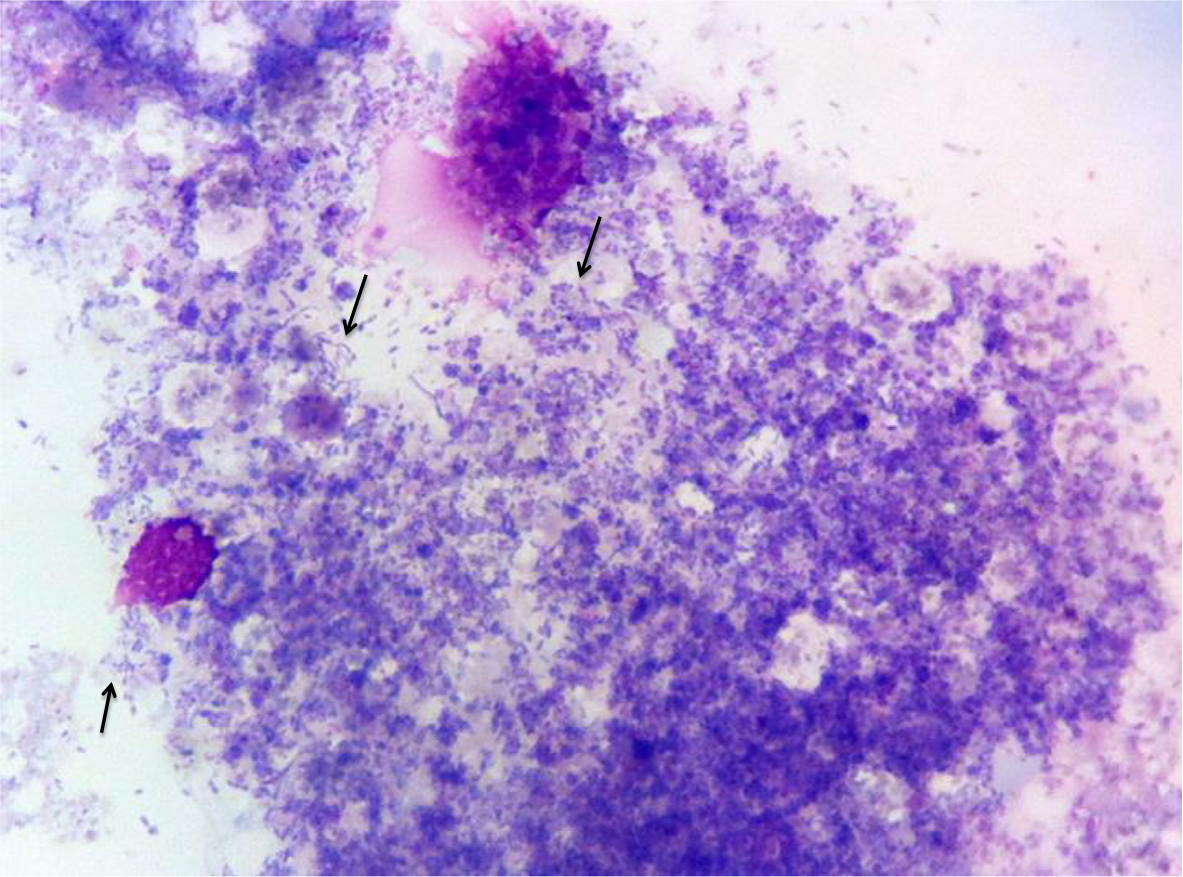
**AAE2 cells at 6 weeks post-inoculation.** Photomicrograph of cytocentrifugation preparation of infected AAE2 cells. The cells contain abundant, pleomorphic, rod-shaped bacteria (Arrows). (Wright-Giemsa, 100X magnification) Images were captured with Mioticam 580 with 5.0 megapixel (Motic North America, British Columbia, Canada).

**Figure 3:**
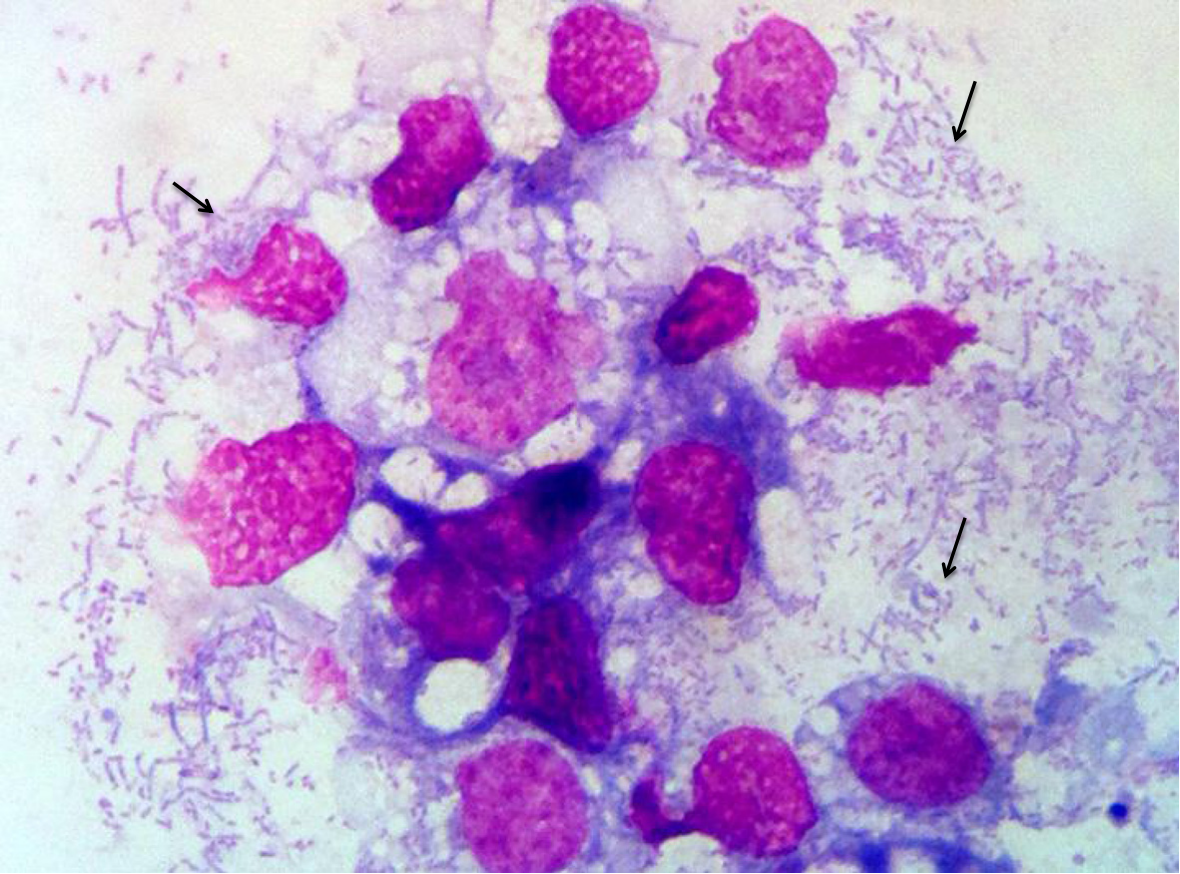
**ISE-6 cells at 6 weeks post inoculation.** Photomicrograph of cytocentrifugation preparation of infected ISE-6 cells. The cells contain abundant, pleomorphic, rod-shaped bacteria (Arrows). (Wright-Giemsa stain, 100X magnification) Images were captured with Mioticam 580 with 5.0 megapixel (Motic North America, British Columbia, Canada).

Rickettsia-specific 17-kD qPCR was performed to monitor culture supernates beginning at approximately 4-weeks post-inoculation. At this time pleomorphic rods typical of SFG rickettsiae were visible in both ISE6 and AAE2 cell lines using Wright-Giemsa-stained cytocentrifugation preparations (Figures 2 and 3). Organisms remained visible by microscopy in both cell lines and remained qPCR-positive through over 20 passages. The cell infection rate remained stable at approximately 85% of host cells, with many cells rupturing because of the high numbers of rickettsial organisms within them. Amplification of all three Rickettsial genes (*glt*A, *dsb*, and *romp*A) verified that the infecting *Rickettsia* sp. was *Rickettsia amblyommii*. Obtained sequences from PCR were identical to those described above. Analysis of *romp*A clones generated with primers 107F and 299R by megaBLAST revealed 98–100% identity with *Candidatus Rickettsia amblyommii* strain GAT-30 V (GenBank:NR 074471.1 and GenBank:CP003334.1). No other bacteria species were detected in culture, even when clones were prepared and sequenced from amplicons generated by bacteria-wide primers. Further genetic analysis is needed to compare this new American isolate with the GAT-30 V strain to determine whether regional differences in gene sequences and protein translations might exist.

## Conclusions

The first successful cultivation and propagation of *R. amblyommii* in both *Ixodes* and *Amblyomma* tick cell lines and the second isolation of the organism in the United States are reported here. *A. americanum* is the most common tick infesting people in the central, southern, and southeastern United States, and, as has been shown in previous studies, the majority of adult LSTs are infected with *R. amblyommii*. Attempts to isolate *R. amblyommii* from clinically ill patients and wildlife are ongoing.

## Abbreviations

SFG: Spotted fever group; LST: Lone star tick, common name for *Amblyomma americanum*.

## Competing interests

The authors declare that they have no competing interests.

## Authors’ contributions

KS designed the molecular experiments, participated in field work, performed the tick work, monitored the cultures by PCR and drafted the manuscript; HW designed the culture experiments and assisted in drafting the manuscript; MP maintained the tick cell culture and edited the manuscript; AB assisted in the interpretation of the data and revised the final manuscript; AR participated in field work and assisted in data interpretation. All authors read and approved the final version of the manuscript.
